# Cardiorespiratory fitness assessment and prediction of peak oxygen consumption by Incremental Shuttle Walking Test in healthy women

**DOI:** 10.1371/journal.pone.0211327

**Published:** 2019-02-07

**Authors:** Liliana Pereira Lima, Hércules Ribeiro Leite, Mariana Aguiar de Matos, Camila Danielle Cunha Neves, Vanessa Kelly da Silva Lage, Guilherme Pinto da Silva, Gladson Salomão Lopes, Maria Gabriela Abreu Chaves, Joyce Noelly Vitor Santos, Ana Cristina Resende Camargos, Pedro Henrique Scheidt Figueiredo, Ana Cristina Rodrigues Lacerda, Vanessa Amaral Mendonça

**Affiliations:** 1 Programa de Pós-Graduação em Reabilitação e Desempenho Funcional, Departamento de Fisioterapia, Universidade Federal dos Vales do Jequitinhonha e Mucuri, Diamantina, Minas Gerais, Brasil; 2 Laboratório de Inflamação e Metabolismo – LIM, CIPq Saúde, Universidade Federal dos Vales do Jequitinhonha e Mucuri, Diamantina, Minas Gerais, Brasil; UFMG, BRAZIL

## Abstract

**Introduction:**

Preliminary studies have showed that the Incremental Shuttle Walking Test (ISWT) is a maximal test, however comparison between ISWT with the cardiopulmonary exercise test (CEPT) has not yet performed in the healthy woman population. Furthermore, there is no regression equation available in the current literature to predict oxygen peak consumption (VO_2_ peak). Thus, this study aimed to compare the ISWT with CEPT and to develop an equation to predict peak oxygen uptake (VO_2_ peak) in healthy women participants.

**Methods:**

First, the VO_2_ peak, respiratory exchange ratio (R peak), heart rate max (HR max) and percentage of predicted HR max (% predicted HR max) were evaluated in the CEPT and ISWT (n = 40). Then, an equation was developed to predict the VO_2_ peak (n = 54) and its validation was performed (n = 20).

**Results:**

There were no significant differences between the ISWT and CEPT of VO_2_ peak, HR max and % predicted HR max values (P>0.05), except for R peak measure in the ISWT (1.22 ± 0.13) and CEPT (1.18 ± 0.1) (P = 0.022). Therefore, both tests showed a moderate positive correlation of VO_2_ peak (r = 0.51; P = 0.0007), HR max (r = 0.65; P<0.0001) and R peak (r = 0.55; P = 0.0002) and the Bland-Altman analysis showed agreement of VO_2_ peak (bias = -0.14). The distance walked on ISWT and age explained 36.3% (R^2^ Adjusted = 0.363) of the variance in VO_2_ peak. The equation developed was VO_2_ peak (predicted) = 19.793 + (0.02 x distance walked)—(0.236 x age). There was no statistically significant difference between the VO_2_ peak measured directly and the predicted, and the Bland-Altman analysis showed agreement (bias = 1.5 ml/kg/min).

**Conclusion:**

ISWT is a maximal test showing similar results compared to the CEPT, and the predicted equation was valid and applicable for VO_2_ peak assessing in young adult healthy women.

## Introduction

Cardiorespiratory fitness (CRF) is defined as the ability to sustain dynamic exercise by large muscle groups over time at moderate to high intensity levels [1]. Furthermore, CRF has been used to measure exercise capacity and provide information about physical limitation, morbidity prognosis, and responsiveness to treatment [2]. The current gold standard for the evaluation of CRF is the direct measurement of maximal oxygen uptake (VO_2_max) which represents the maximal achievable level of oxidative metabolism involving large muscle groups [3]. However, in clinical testing situations, the exercise usually is limited by symptoms before the individual achieve the VO_2_max. Consequently, VO_2_ peak is often used as an estimate for VO_2_max and they are used interchangeably [3].

The laboratory assessment of CRF through maximal tests on treadmills or cycle ergometers (cardiopulmonary exercise testing-CEPT) has a high cost [4] and require specialized professionals and equipments that is not always available [5]. Thus, field tests were developed and have been increasingly used in clinical practice, such as the Six-minute walk test and the Incremental Shuttle Walking Test (ISWT). ISWT was created by Singh et al. [6] to assess the CRF of patients with chronic pulmonary obstructive disease (COPD) and later used in other conditions or healthy subjects [7, 8, 9, 10, 11].

Several studies had already shown strong correlations between the performance on CEPT and ISWT [5, 12, 13, 14]. Some studies have showed that the ISWT is a maximal test in the pediatric and elderly population [15, 16, 17, 18], however the intensity of ISWT was often indirectly assessed by predictive equations [15, 16, 17]. Hence, our study group compared cardiorespiratory responses between ISWT and CEPT in healthy young adult men [14] and adolescent boys (data not published), where the results showed moderate to high significant correlation and agreement, concluding that the ISWT is a maximal test in these subjects. In addition, a VO_2_ peak prediction equation based on ISWT variables was developed and it demonstrated feasibility and validity [14]. However, this study did not include women in the assessments, remaining a gap in the literature about the ISWT in healthy women.

In this paper, we evaluate the CRF in healthy young women by comparing and correlating VO_2_ peak, respiratory quotient peak (R peak), maximum heart rate (HR max) and percentage of predicted maximum heart rate (% predicted HR max), between ISWT with CEPT through direct analysis of the exhaled gases, aiming to classify the ISWT intensity and to elaborate a predictive equation to estimate the VO_2_ peak in young adult women population.

## Materials and methods

### Subjects

Women between 18 and 45 years of age were recruited by convenience from Diamantina city, Minas Gerais state, Brazil. The inclusion criteria were: self-report of no acute or chronic diseases; eutrophic according to the body mass index (BMI between 18.5 and 24.9 kg/m^2^); no smoker; sedentary (not performing physical activity for 30 minutes or more at least three times a week) [19]. The participants were excluded from the study if did not reach the maximal test values on the treadmill (% predicted HR max higher than 90%) and those who failed to understand the tests. This study was approved by the Ethics and Research Committee of Universidade Federal dos Vales do Jequitinhonha e Mucuri, Brazil (protocol 1.184.419/2015) and conducted in accordance with the Resolution N° 466/12 of the National Health Council and the Declaration of Helsinki. The participants were informed about the procedures and potential risks associated with the study and all gave written informed consent.

### Stages of the study

This was a cross-sectional study divided into three stages: (1) To compare the CEPT and the ISWT and evaluate the correlation and agreement between the variables VO_2_ peak, R peak, HR max and % predicted HR max, as well as determine the ISWT intensity in the female population; (2) To elaborate an equation to predict the VO_2_ peak; and (3) validate this equation. The sample size was calculated using the statistical program G.Power 3.1 and was based on the number of variables to be included in the multiple regression analysis and the minimum number of observations required. Considering an effect size of 0.68 and power of 0.99, 54 volunteers were required in order to develop a linear model including up to four variables [14]. To validate the equation, another 20 volunteers were required [14].

To evaluate the cardiorespiratory fitness, all participants were instructed to avoid physical activity and intake caffeine and alcohol in the 24 h prior to the test, to get at least 8 hours of sleep the night before, to eat a light meal and to ingest 500 ml of water two hours before the tests [19]. During all tests performed, the exhaled gases were collected and assessed by a portable telemetric gas analysis system (K4b2, Cosmed, Rome, Italy). Among other variables, VO_2_, R and HR breath-by-breath were monitored. The data were tabulated and was defined as VO_2_ peak and R peak the highest value of these measures at peak effort [20]. Predicted HR max was calculated by the equation HR max = 220 –age [21].

The first stage of the study was performed on three consecutive days. On the first day, the anthropometric variables weight, height and BMI, were measured and a familiarization was performed. On subsequent days, the CEPT or the ISWT was performed by randomization.

The ISWT was performed in a 10-m course identified by two cones placed 0.5 m from each end point, with an initial speed of 0.5 m/s, increasing 0.17 m/s every minute. The protocol used was composed of 15 stages of 1 min, to prevent the ceiling effect [10, 22] and the walking speed was dictated by a sound [6]. The test was interrupted if the volunteer did not reach the cone once, at the request of the volunteer or for some other reported symptom (dyspnea, dizziness, vertigo, and angina). The CEPT protocol was based on the progression of the ISWT, with the same initial speed and the same speed increase every minute, without changing the incline of the treadmill. The criteria for interrupting the CEPT was systolic blood pressure (SBP) greater than 210 mm Hg; diastolic blood pressure greater than 120 mm Hg; sustained decrease in SBP; angina; dyspnea; cyanosis; nausea; dizziness; or by volunteer’s request [19].

In the second and third stage, the participants performed two ISWT with an interval of 30 minutes between then [23] and the results of the test with the longest walking distance were used for the statistical analysis. To validate the equation, a different group of women was selected according to the same inclusion criteria of the study. The VO_2_ peak obtained by the gas analyzer was compared with the VO_2_ peak predicted by the elaborated equation.

### Statistical analysis

Statistical analysis was performed with the Statistical Package for Social Sciences programs version 22.0 (SPSS Inc., Chicago, IL, USA) and GraphPad Prism 5.0 (Inc., USA). Data were presented as mean (standard deviation). In the first stage the normality of the data was calculated by Shapiro-Wilk test. As the data presented normal distribution, the comparison between the means of the physiological variables evaluated (VO_2_ peak, R peak, HR max and % predicted HR max) were performed using Paired T-test. The correlation analysis of the variables collected was performed by Pearson`s correlation. The agreement of the variables collected was performed by the Bland-Altman analysis. In the second stage, the Kolmogorov-Smirnov test was used, and the analysis of multiple linear regression was performed with the variables age, weight, height and distance walked defined a priori to elaborate the VO_2_ peak prediction equation. For the validation of the equation, the Shapiro-Wilk test was performed and then the paired T-test to compare the mean values of the VO_2_ peak values obtained by the equation with those obtained by the analyzer of gases. In addition, the comparison between the women of first and third stages were realized using the Independent test t or Mann-Whitney test, according of normality of data. The level of statistical significance adopted was P <0.05.

## Results

### First stage: Comparison between CEPT and ISWT

The general characteristics of the participants of first and second stage and their performance on ISWT are showed in Table 1.

**Table 1:** General characteristics of participants study.

Variable	*N = 54*
Age (years)	26.41± 5.6 (24.89–27.92)
Weight (kg)	56.56 ± 9.1 (54.08–59.05)
Height (m)	1.63 ± 0.1 (1.608–1.641)
BMI (kg/m^2^)	21.86 ± 1.8 (21.38–22.33)
Distance walked (m)	821.10 ± 118.9 (788.7–853.6)
Walking speed (m/s)	2.06 ± 0.2 (2.013–2.104)

The data is presented as mean ± SD (95% CI). BMI = body mass index.

Forty volunteers performed both ISWT and CEPT and their cardiorespiratory responses are presented in Table 2. There was no statistically significant difference for any of the variables, except for the R peak, which was higher in the ISWT. According to the percentage of predicted HR max (above 90%) and R peak (> 1.1), the ISWT could be considered a test of maximum intensity [14, 24, 25]. Blood pressure and heart rate were monitored during all tests and there were no intercurrences.

**Table 2:** Comparison between the cardiorespiratory variables obtained in the ISWT and in the CEPT.

Outcome	ISWT (n = 40)	CEPT (n = 40)	P-value
VO_2_ peak (mL/kg/min)	30.20(4.78)	30.35(4.81)	0.842
R peak	1.22(0.13)	1.18(0.1)	0.022*
HR max (bpm)	187.6(9.26)	186.7(9.63)	0.460
Predicted HR max (%)	97.25(4.51)	96.77(4.69)	0.463

The data is presented as mean (SD).

* P<0.05.

ISWT = Incremental Shuttle Walking Test; CEPT = cardiopulmonary exercise test; VO_2_ = oxygen uptake; R = respiratory exchange ratio; HR = heart rate; Paired-t test.

**Fig 1:**
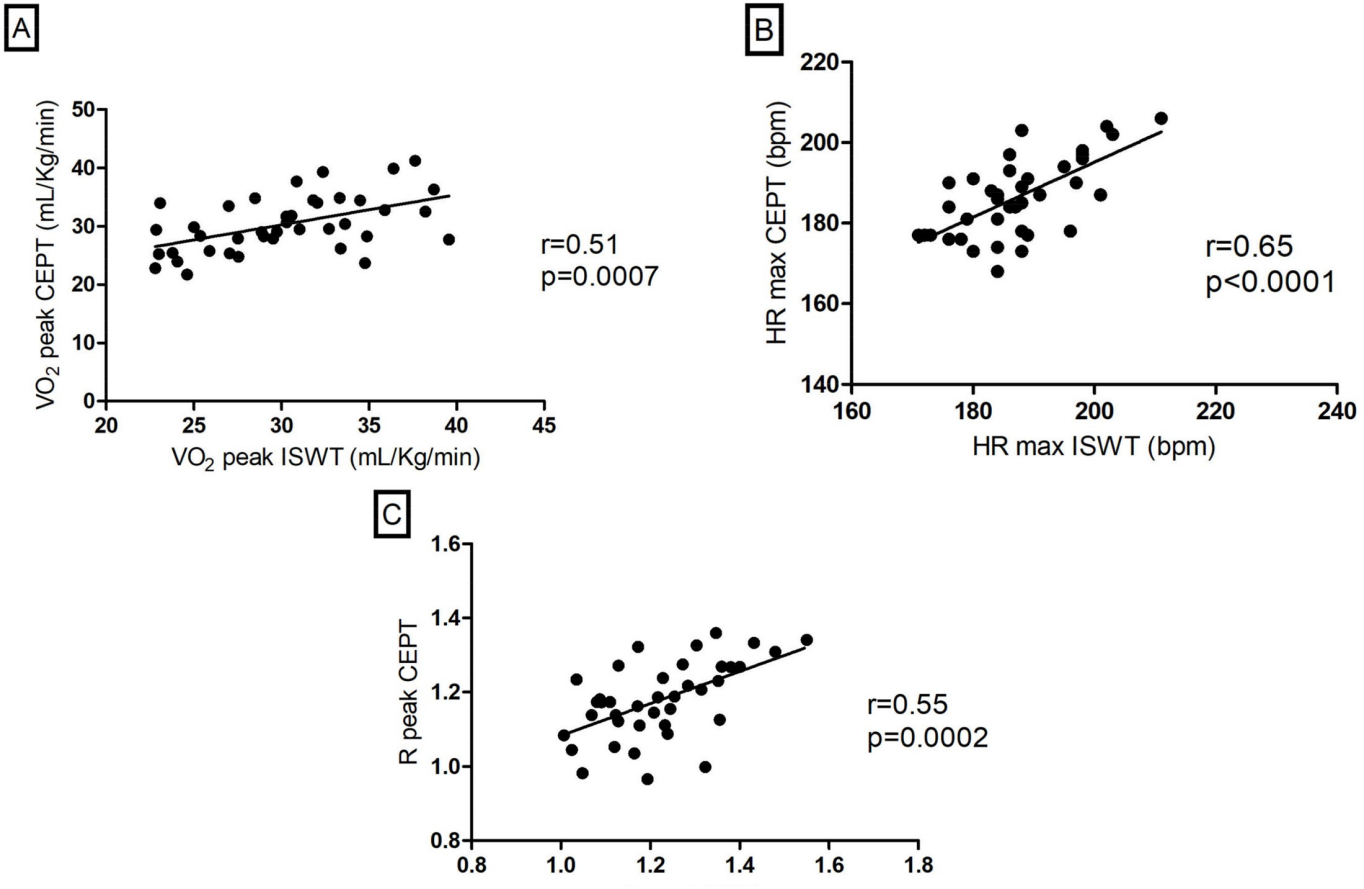
Correlation between (A) VO_2_ peak, (B) HR max and (C) R peak in the ISWT and the CEPT. ISWT = Incremental Shuttle Walking Test; CEPT = cardiopulmonary exercise test; VO_2_ = oxygen uptake; HR max = maximum heart rate; R = respiratory exchange ratio.

**Fig 2:**
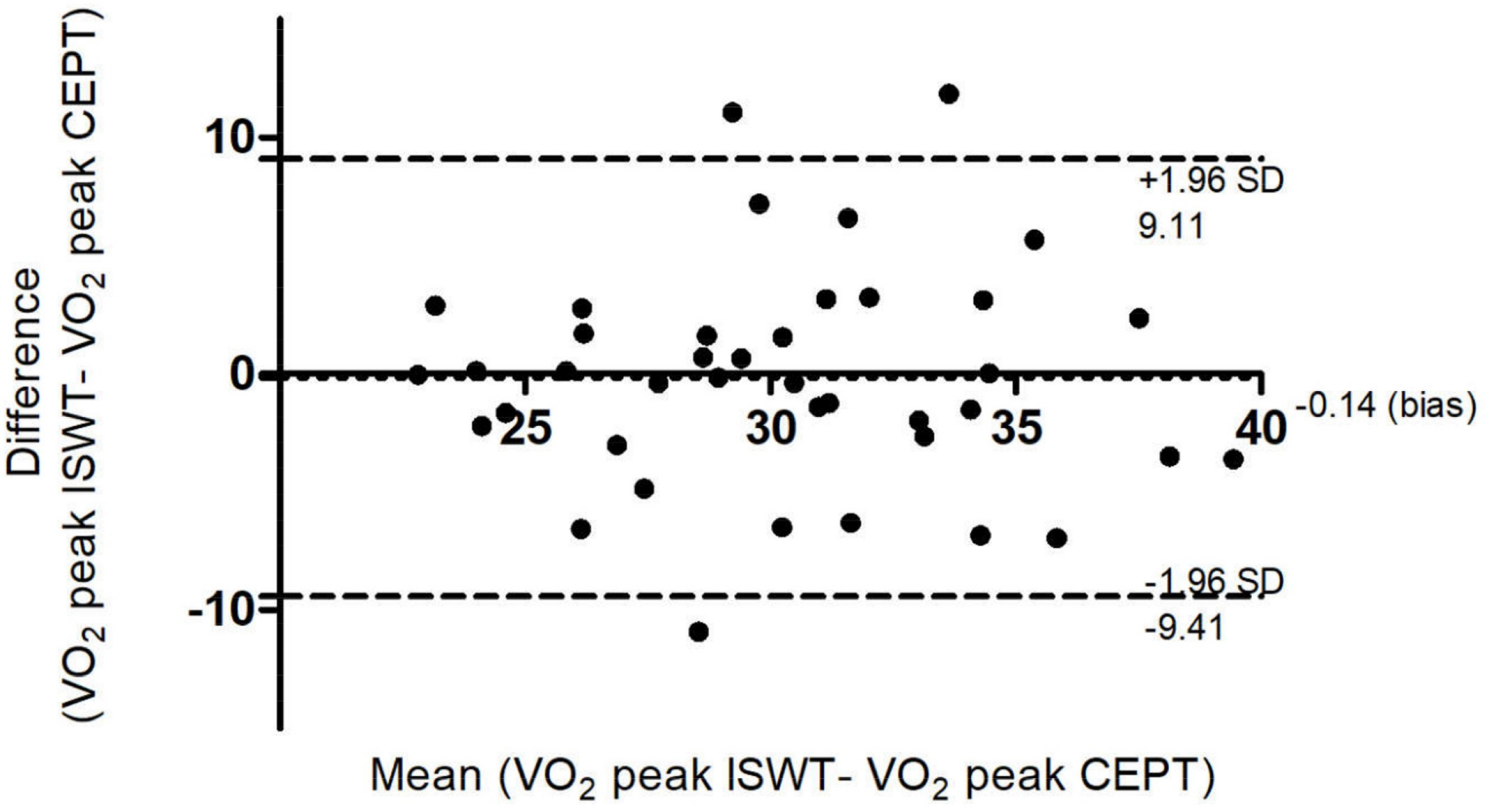
Bland-Altman agreement of VO_2_ peak in the ISWT and the CEPT. ISWT = Incremental Shuttle Walking Test; CEPT = cardiopulmonary exercise test; VO_2_ = oxygen uptake.

Significant correlations were found for the variables VO_2_ peak, HR max and R peak (Fig 1). The Bland-Altman analysis also demonstrated agreement between the VO_2_ peak in the ISWT and in the CEPT (Fig 2).

### Second stage: Reference equation for VO_2_ peak

The univariate analysis was performed with the variables age, weight, height and distance walked (N = 54). A model of stepwise linear multiple regressions showed distance walked on ISWT and age explained 36.3% (Adjusted R Square = 0.363) of the variance in VO_2_ peak and this was significant (p = 0.014). The reference equation for the VO_2_ peak in the ISWT was:

$$VO_{2\;\;}peak\left(predicted\right)=19.793+\left(0.02\;x\;distance\;walked\right)-\left(0.236\;x\;age\right)$$

Performing a posthoc analysis of the linear multiple regression model, the power of analysis was 0.99.

### Third stage: Validation of the reference equation

The characteristics of the volunteers who participated in the equation validation are present in Table 3.

**Table 3:** General characteristics of the study participants.

Variable	*N = 20*
Age (years)	25.85 ± 5.6 (23,24–28,46)
Weight (kg)	55.84 ± 5.7 (53.16–58.51)
Height (m)	1.62 ± 0.04 (1.594–1.638)
BMI (kg/m^2^)	21.34 ± 1.5 (20.61–22.07)
Distance walked (m)	865 ± 100.2 (818.1–911.9)
Walking speed (m/s)	2.11 ± 0.14 (2.049–2.181)

The data is presented as mean ± SD (95% CI). BMI = body mass index.

There was no statistically significant difference between the participants of equation elaboration and validation for age, weight, height, BMI, Distance walked and Walking speed (P>0.05; data not show).

**Fig 3:**
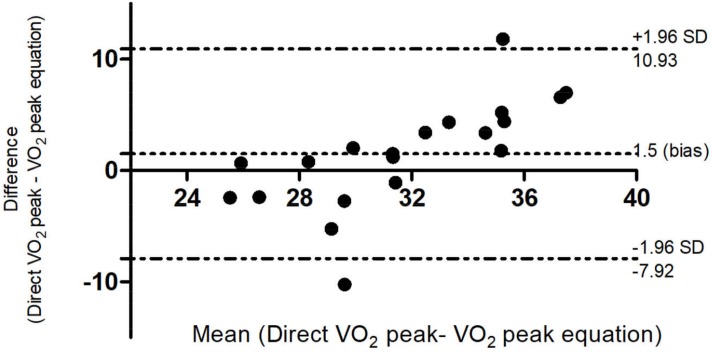
Bland-Altman agreement of VO_2_ peak in the validation of the reference equation. ISWT = Incremental Shuttle Walking Test; CEPT = cardiopulmonary exercise test; VO_2_ = oxygen uptake.

When the reference equation was applied in this group, there was no statistically significant difference of the VO_2_ peak obtained by the use of the gold standard method in comparison to that obtained by the equation [32.50 (5.6) mL/kg/min and 30.99 (2.6) mL/ kg/ min, respectively; p = 0.178)]. It was possible to verify the agreement between these measures by the Bland-Altman method, in which a bias of 1.5 mL/kg/min was observed, representing a difference of 3.6% between the ways of measuring VO_2_ peak (Fig 3).

## Discussion

In the present study it was observed that the direct VO_2_ peak measurement was concordant between the CEPT and the ISWT and that ISWT was a maximum test in the young healthy women population. Considering that the direct analysis of VO_2_ is not feasible for clinical practice, an equation was elaborated and validated to estimate this measure. The distance walked on ISWT and age were the variables that composed the equation. Furthermore, there was agreement between VO_2_ peak measured directly and that estimated by the elaborated equation, indicating its validity and applicability in this population.

In the last years the ISWT has been applying in the healthy population [10, 11, 18, 26]. However, as far as we know, this is the first study that a comparison between CEPT and ISWT was performed in healthy young women. Initial investigations were carried out in patients with COPD, cystic fibrosis and chronic heart failure, showing strong and significant correlations for VO_2_ peak of CEPT and ISWT [7, 9, 12].

In a study recently published by our research group, male healthy adults showed HR max, VO_2_ peak and R peak values with strong and significant correlations and agreement between the ISWT and the CEPT, with ISWT being a maximal test for this population [14]. Considering that the maximum VO_2_ values for women are about 70% of the average values for men [27] and that is not known whether ISTW is a maximum test for healthy young women, we initially investigated the intensity of ISTW.

Since the values of HR max above 90% of predicted and R peak > 1.1 [14, 24, 25], we establish that this is a maximum test for this population, and similar VO_2_ peak results were found between CEPT and ISWT. Further tests carried out with patients with cardiopulmonary diseases concurred with our findings [6, 12, 28, 29]. However, data on the validity of the ISWT to evaluate VO_2_ peak in healthy individuals are scarce in the literature [18]. Gonçalves et. al [30], studying subjects of both sexs, different age (≥ 18 years old), who presented comorbidities such as arterial hypertension, peripheral vascular disease, arthritis and cardiopathies, also concluded that ISWT above 12 levels requires maximum effort in these individuals.

As the direct analysis of the exhaled gases has a high cost, the use of prediction equations becomes more applicable due to the feasibility and low cost. Considering our results that ISTW is a maximum test to healthy women, its usefulness is reinforced as a simple way of measuring CRF. In this context, an equation was then elaborated for the prediction of VO_2_ peak in ISTW.

In our study, age and distance walked accounted for more than 30% of VO_2_ peak variance. In the literature it is reported that beyond gender, other factors that influence VO_2_ peak as genetic factors, age, weight, and training [31]. Findings similar to our study were found in obese women, where there was a significant correlation between the VO_2_ peak in the cardiopulmonary exercise test with the ISWT VO_2_ peak and the ISWT distance [5]. In this same study, the variables age and distance walked by the ISWT explained the predictive model for the VO_2_ peak.

Only two other studies have published a reference equation for VO_2_ peak using ISWT, highlighting the variables distance and body mass in the prediction [11, 32]. In the study of Dourado et. al [11] the distance in the ISWT was selected, the maximum walking velocity, and distance in the ISWT × body mass as the only determinants of the peak VO_2_. This is consistent with the variables selected in our study. However, they did not compare to another cardiopulmonary exercise test, nor did they validate the equation.

As age is a determining factor for VO_2_ peak, it is important to highlight that several studies have used the ISWT in the older population [10, 11, 22, 23, 26] or in children and adolescents [15–17], and some evaluated stratifying age groups [2, 30]. Due to the influence of cardiorespiratory fitness on functional independence, there is great interest in describing age-related changes in maximum oxygen consumption. Evidences support a 10% per decade decline in VO_2_ max in men and women regardless of activity level [33]. For all the facts reported, it makes sense for age to be a predictor of VO_2_ peak in the elaborated equation.

Our study presents differentials when proposing a prediction equation for VO_2_ peak, the main variable for evaluation of cardiorespiratory fitness [19, 34], since most of the studies with ISWT focus on the prediction of walking distance [2, 10, 15, 17, 22, 23]. In addition, those who did the VO_2_ peak prediction equation for women did not validate it [5, 11]. The equation developed in this study was validated in other volunteers and the VO_2_ peak values obtained by the equation and the values of VO_2_ peak obtained by the gas analyzer were similar, indicating that the application of the equation is feasible to estimate the VO_2_ peak of the chosen population.

The limitation of the study was the level of physical activity having been self-reported, but this strategy is adopted in scientific studies [35, 36].

## Conclusion

The Incremental Shuttle Walking Test was concordant with the CEPT, requiring maximum effort in young health women. The elaborated equation is valid and applicable, being a simple and inexpensive tool to evaluate the cardiorespiratory fitness in the study population.
